# Supplementary material for: The social stratification of internal migration and daily mobility during the COVID-19 pandemic
Source: Sci Rep. 2024 May 27;14:12140. doi: 10.1038/s41598-024-63098-5 (PMC11130293; doi:10.1038/s41598-024-63098-5)
Supplement: Supplementary file 1 — Supplementary Information. [file 41598_2024_63098_MOESM1_ESM.pdf]

The social stratification of internal migration and daily mobility during the COVID-19 pandemic

Erick Elejalde[1*†], Leo Ferres[2,3,4*†], Victor Navarro[2,3], Loreto Bravo[2,3], Emilio Zagheni[5]

[1]L3S Research Center, Leibniz University Hannover, Hannover, Germany.
[2]Institute of Data Science, Universidad del Desarrollo, Santiago, Chile.
[3]Telefónica R&D, Telefónica, Santiago, Chile.
[4]ISI Foundation, Torino, Italy.
[5]Max Planck Institute for Demographic Research, Rostock, Germany.

*Corresponding author(s). E-mail(s): elejalde@l3s.uni-hannover.de; lferres@udd.cl;
†These authors contributed equally to this work.

Appendix A Daily mobility for comunas in Santiago

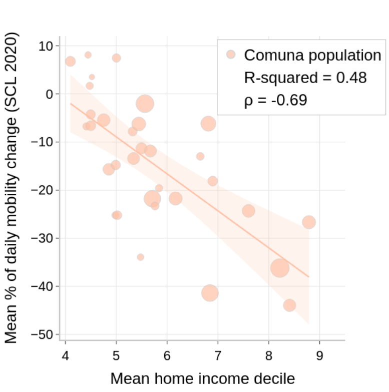

(a) IM change 2020

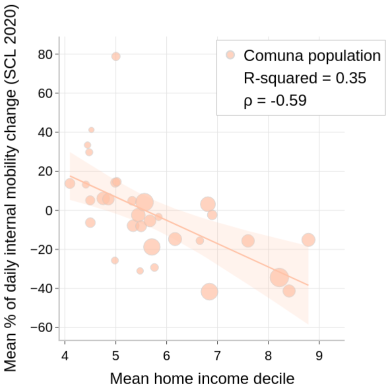

(b) IM internal change 2020

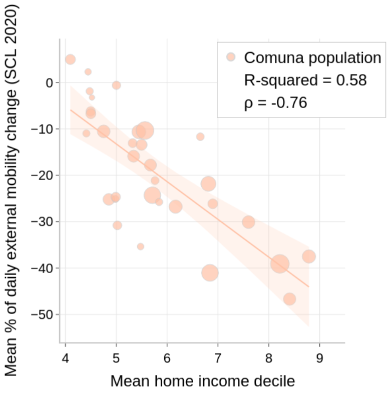

(c) IM external change 2020

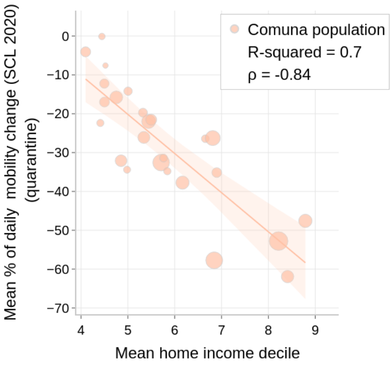

(d) IM change 2020 (for quarantine periods)

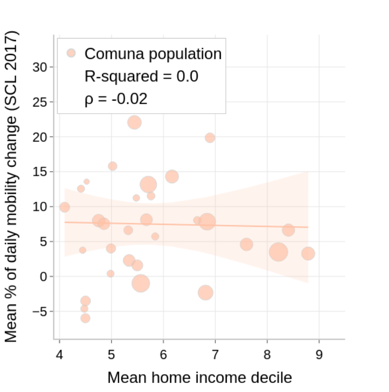

(e) IM change 2017

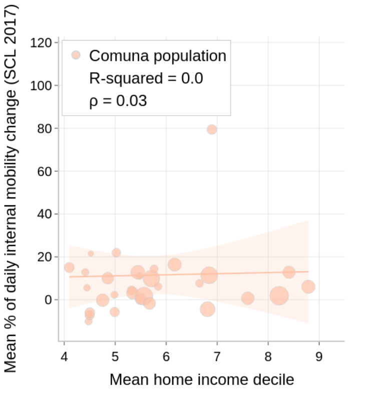

(f) IM internal change 2017

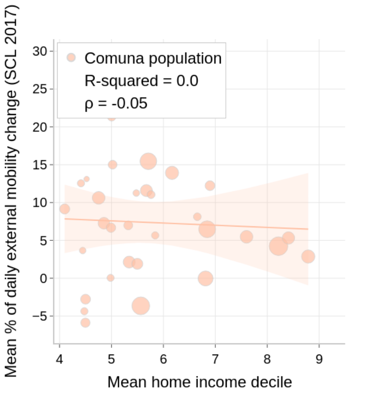

(g) IM external change 2017

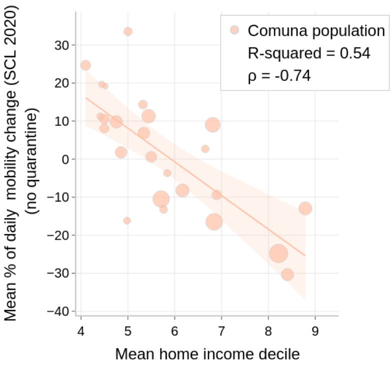

(h) IM change 2020 (for non quarantine periods)

Fig. A1: Daily Mobility Index for comunas in SCL

Appendix B Correlation between National Census 2017 and Internal Migration Mobile Model

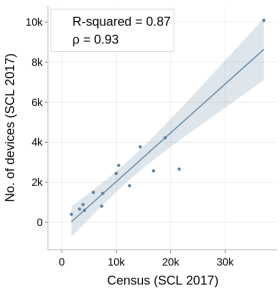

(a) Immigration - Regions of Origin

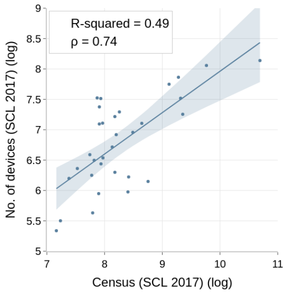

(b) Immigration - Comunas of Destination

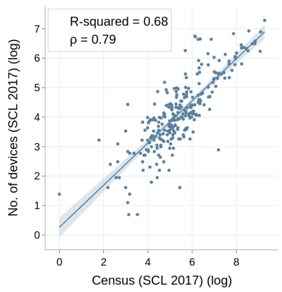

(c) Immigration - Comunas of Origin

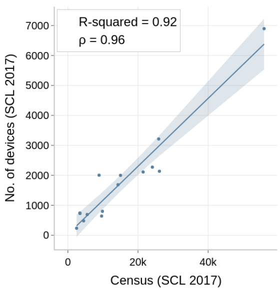

(d) Emigration - Regions of Destination

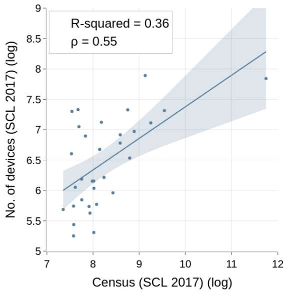

(e) Emigration - Comunas of Origin

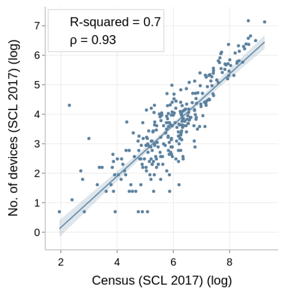

(f) Emigration - Comunas of Destination

Fig. B2: Comparing the movement based on the Internal Migration Mobile Model (Mar.-Nov., 2017) and the movement reported by the National Census 2017. B2a and B2d represent movement between other regions and the Metropolitan Region (MR). B2b and B2e represent movement into and from comunas in the MR. B2c and B2f represent the migration between the MR and other comunas outside this region.
